# Supplementary material for: Novel and Simple Method for Quantification of 2,4,6-Trichlorophenol with Microbial Conversion to 2,4,6-Trichloroanisole
Source: Microorganisms. 2023 Aug 23;11(9):2133. doi: 10.3390/microorganisms11092133 (PMC10535749; doi:10.3390/microorganisms11092133)
Supplement: Supplementary file 1 [file microorganisms-11-02133-s001.zip › microorganisms-2533719-SI.pdf]

Supplementary Materials

Table S1 Detailed information on the fungi obtained in this study.

No.	Sample origin		Appearance		2,4,6-TCA productivity *	Sequence of ITS region	
	dates	Location	Colony Color	filamentous fungi		Identified genus	Registered accession no.
0521-K05	21-May-2021	T1	Yellow and white	+	-	*Aspergillus*	OQ657949
0521-K08	21-May-2021	T1	Light brown	-	-	*Cutaneotrichosporon*	OQ657950
0521-K09	21-May-2021	T1	White	+	-	*Geotrichum*	OQ657951
0521-K11	21-May-2021	T1	White	-	-	*Apiotrichum*	OQ657952
0521-K13	21-May-2021	T1	Pink	-	-	*Rhodotorula*	OQ657953
0521-K14	21-May-2021	T1	Brown, sky blue and white	+	+ +	*Penicillium*	OQ657954
0521-K15	21-May-2021	T1	White	+	-	*Apiotrichum*	OQ657955
0521-K16	21-May-2021	T1	White	+	+ +	*Trametes*	OQ657956
0521-K17	21-May-2021	T1	White	+	-	*Trametes*	OQ657957
0623-K03	20-Jun-2021	T2	White	-	-	*Candida*	OQ657958
0623-K04	20-Jun-2021	T2	Pale orange	-	-	*Trichosporon*	OQ657959
0623-K05	20-Jun-2021	T2	Pink	-	-	*Rhodotorula*	OQ657960
0623-K08	20-Jun-2021	T2	Dark green	+	-	*Cladosporium*	OQ657961
0729-K06	28-Jul-2021	T2	White	+	-	*Trametes*	OQ657962
0729-K12	28-Jul-2021	T2	Pink	-	-	*Rhodotorula*	OQ657963
0928-K02	28-Sep-2021	T3	Pale orange	-	-	*Cutaneotrichosporon*	OQ657964
0928-K03	28-Sep-2021	T3	Yellow green and white	+	+ +	*Trichoderma*	OQ657965
0928-K04	28-Sep-2021	T3	Light brown and light blue	+	+ +	*Penicillium*	OQ657966
0928-K06	28-Sep-2021	T3	Pale orange	-	+	*Apiotrichum*	OQ657967
0928-K07	28-Sep-2021	T3	Yellow	+	+	*Aspergillus*	OQ657968
0928-K08	28-Sep-2021	T3	Bright yellow	+	+	*Aspergillus*	OQ657969
0928-K09	28-Sep-2021	T3	White	+	+ +	*Trichoderma*	OQ657970
0928-K11	28-Sep-2021	T3	Dark red	+	-	*Talaromyces*	OQ657971

0928-K12	28-Sep-2021	T3	Light yellow, pink and white	+	-	*Penicillium*	OQ657972
1018-K01	18-Oct-2021	B1	Yellow green	+	+	*Cladosporium*	OQ657973
1018-K02	18-Oct-2021	B1	Dark green and white	+	-	*Cladosporium*	OQ657974
1116-K02	16-Nov-2021	R1	Pale pink	-	-	*Naganishia*	OQ657975
1116-K03	16-Nov-2021	R1	White	-	-	*Hanseniaspora*	OQ657976
1116-K04	16-Nov-2021	R1	Orange	+	+ +	*Talaromyces*	OQ657977
1122-K01	20-Nov-2021	R2	Yellow green and white	+	+ +	*Trichoderma*	OQ657978
1126-K01	26-Nov-2021	B2	Pale orange	-	-	*Naganishia*	OQ657979
1126-K02	26-Nov-2021	B2	Dark pink	-	-	*Rhodotorula*	OQ657980

*: TCA productivity in the table was shown as negative (-) for strains with no confirmed TCA productivity, positive (+) for strains with confirmed TCA productivity, and positive plus (++) for strains with relatively high TCA productivity.

Table S2 Detailed information on the bacteria obtained in this study.

No.	Sample origin		Colony Color	2,4,6-TCA productivity*	16S rRNA sequence	
	dates	Location			Identified genus	Registered accession no.
1018-S01	18-Oct-2021	B1	Dark yellow	++	*Sphingomonas*	OQ651227
1018-S02	18-Oct-2021	B1	Pale yellow	-	*Micrococcus*	OQ651203
1018-S03	18-Oct-2021	B1	White	++	*Mycolicibacterium*	OQ651228
1018-S04	18-Oct-2021	B1	White	++	*Mycolicibacterium*	OQ651229
1018-S05	18-Oct-2021	B1	Transparent	-	*Pelomonas*	OQ651230
1116-S01	16-Nov-2021	R1	White	+	*Acinetobacter*	OQ651204
1116-S03	16-Nov-2021	R1	White	-	*Flavobacterium*	OQ651205
1116-S04	16-Nov-2021	R1	Yellow	-	*Sphingomonas*	OQ651206
1116-S05	16-Nov-2021	R1	Ocher	-	*Flavobacterium*	OQ651207
1116-S06	16-Nov-2021	R1	White	-	*Bacillus*	OQ651208
1116-S07	16-Nov-2021	R1	Orange	-	*Exiguobacterium*	OQ651209
1116-S08	16-Nov-2021	R1	Transparent orange	-	*Rheinheimera*	OQ651210
1116-S09	16-Nov-2021	R1	White	-	*Yersinia*	OQ651211
1116-S13	16-Nov-2021	R1	Ocher	-	*Sphingomonas*	OQ651212
1122-S01	20-Nov-2021	R2	Translucent white	-	*Pseudomonas*	OQ651213
1122-S02	20-Nov-2021	R2	Pale yellow	-	*Chryseobacterium*	OQ651214
1126-S02	26-Nov-2021	B2	White	-	*Acinetobacter*	OQ651215
1126-S03	26-Nov-2021	B2	Pink	-	*Acinetobacter*	OQ651216
1126-S04	26-Nov-2021	B2	Dark orange	-	*Sphingomonas*	OQ651217
1126-S05	26-Nov-2021	B2	Dark yellow	-	*Sphingomonas*	OQ651218
1126-S06	26-Nov-2021	B2	Clear white	-	*Mitsuaria*	OQ651219
1126-S07	26-Nov-2021	B2	Orange	-	*Pseudomonas*	OQ651220
0524-S01	16-May-2022	B3	White	++	*Mycolicibacterium*	OQ651221
0524-S02	16-May-2022	B3	Yellow	-	*Staphylococcus*	OQ651231
0524-S05	16-May-2022	B3	White	++	*Mycobacterium*	OQ651232
0524-S08	16-May-2022	B4	Red	-	*Methylobacterium*	OQ651222
0524-S14	16-May-2022	B4	White	++	*Mycolicibacterium*	OQ651233

0704-S19	26-Jun-2022	B5	Orange	+	*Mycolicibacterium*	OQ651234
0704-S20	26-Jun-2022	B5	White	++	*Mycolicibacterium*	OQ651223
0704-S22	26-Jun-2022	B5	White	++	*Mycolicibacterium*	OQ651235
0704-S23	26-Jun-2022	B5	Red orange	++	*Mycolicibacterium*	OQ651236
0704-S24	26-Jun-2022	B5	Orange	++	*Mycolicibacterium*	OQ651237
0704-S28	26-Jun-2022	B5	White	++	*Mycolicibacterium*	OQ651238
0704-S32	26-Jun-2022	B5	Orange	+	*Mycolicibacterium*	OQ651239
0704-S34	26-Jun-2022	B5	White	++	*Mycolicibacterium*	OQ651240

*: TCA productivity in the table were shown as negative (-) for strains with no confirmed TCA productivity, positive (+) for strains with confirmed TCA productivity, and positive plus (++) for strains with relatively high TCA productivity.

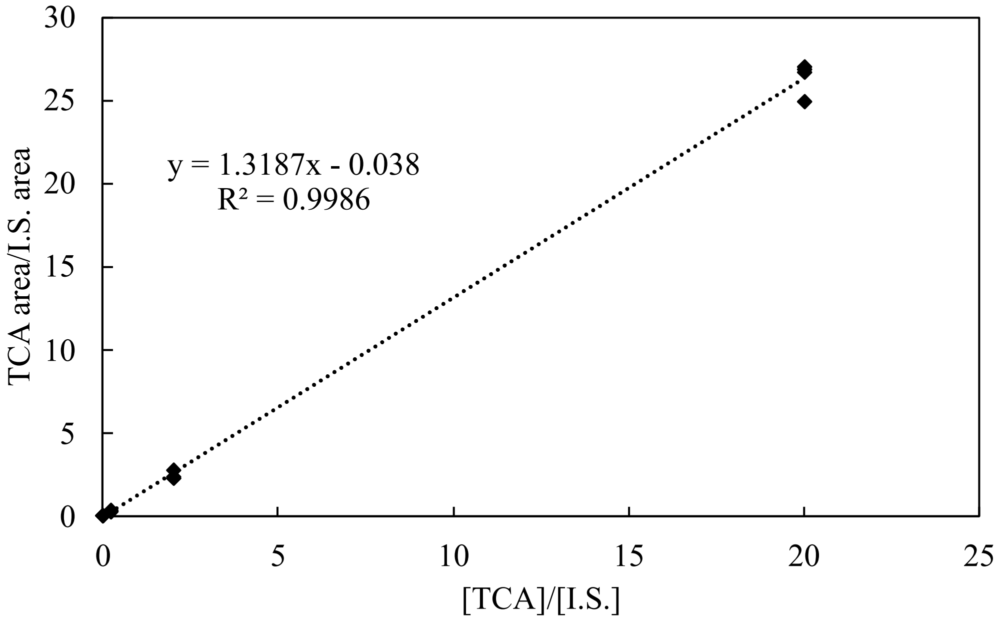

Figure S1 GC-MS calibration curve for TCA.

The calibration was obtained from the measured peak area of TCA vs. TCA concentration in the vial bottle. The peak area and the concentration in the figure were relative values to those of *p*-iodoanisole as internal standard.

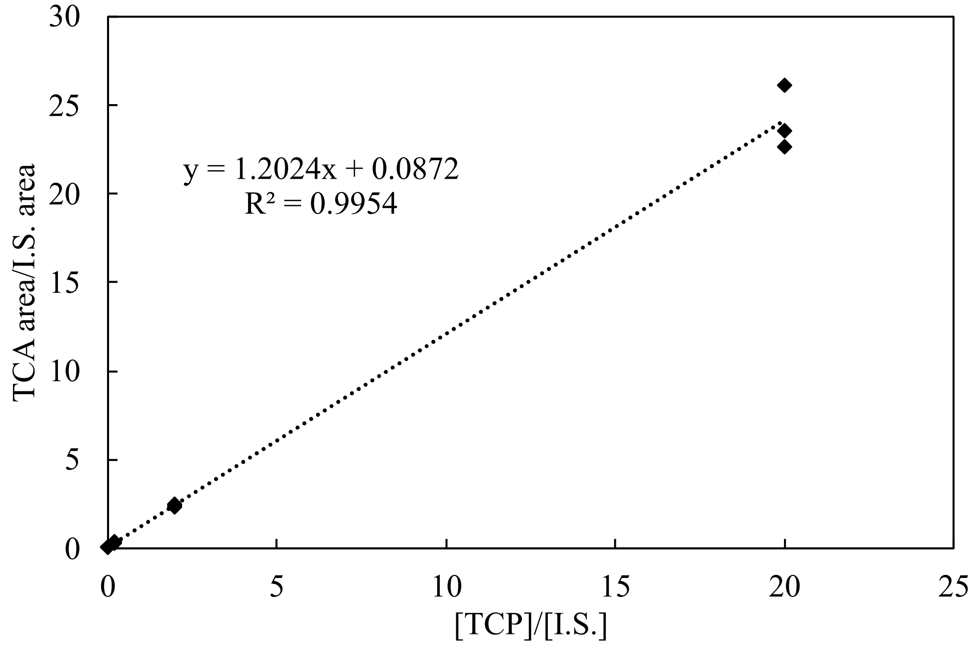

Figure S2 GC-MS calibration curve for TCP.

The calibration was obtained from the measured peak area of TCA vs. TCP concentration added in the vial bottle before biological conversion. The peak area and the concentration in the figure were relative values to those of *p*-iodoanisole as internal standard.

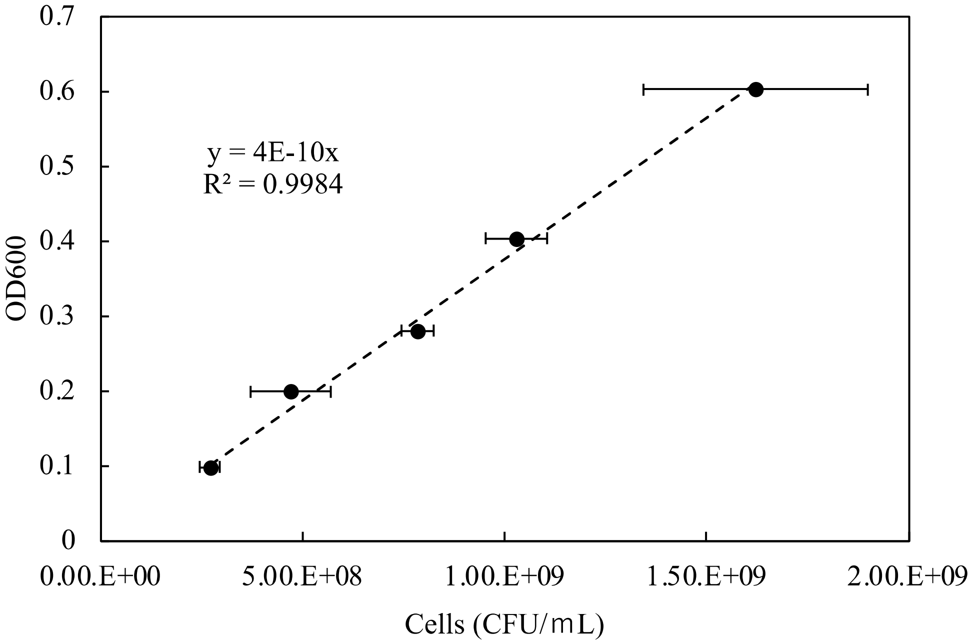

Figure S3 The correlation between colony numbers and the turbidity of the culture medium.
